# Crystal structure of bis­(*N*-*tert*-butyl­benzamidinium) hexa­chlorido­zirconate(IV) di­chloro­methane disolvate

**DOI:** 10.1107/S2056989016003030

**Published:** 2016-03-02

**Authors:** Zhi-Hao Jiang, Jian-Ping Zhao, Xiu-Ming Ma, Sheng-Di Bai

**Affiliations:** a Institute of Applied Chemistry, Shanxi University, Taiyuan 030006, People’s Republic of China

**Keywords:** crystal structure, benzamidinium, zirconate, N—H⋯Cl hydrogen bonds

## Abstract

In the Zr^IV^ complex anion of the title complex salt, [(C_4_H_9_)HNC(C_6_H_5_)NH_2_]_2_[ZrCl_6_]·2CH_2_Cl_2_, the Zr^IV^ cation, located on an inversion centre, is coordinated by six Cl^−^ anions in a distorted octa­hedral geometry with Zr—Cl distances in the range 2.433 (2)–2.4687 (19) Å; in the amidinium cation, the dihedral angle between the aromatic ring and [NCN] plane is 43.3 (4)°. In the crystal, the amidinium cations and [ZrCl_6_]^2−^ anions are linked by N—H⋯Cl hydrogen bonds, forming a two-dimensional network extending along the *b* axis; two di­chloro­methane solvent mol­ecules are linked by a pair of weak C—H⋯Cl hydrogen bonds, forming a centrosymmetric [CHCl]_2_ six-membered ring.

## Abstract 2

In the crystal of the title complex salt, the amidinium cations and the centrosymmetric Zr^IV^ complex anions are linked by N—H⋯Cl hydrogen bonds, forming a two-dimensional network extending along the *b*-axis direction.

## Chemical context   

Amidinates represent an important class in the array of N-centered ligands comparable to the cyclo­penta­dienyl system (Edelmann, 1994 ▸; Barker & Kilner, 1994 ▸; Collins, 2011 ▸). They are four-electron, monoanionic and *N*-donor bidentate chelates, demonstrating a great diversity by variation of substituents on the conjugated N–C–N backbone. Their steric and electronic properties are easily tunable to meet the requirements of different metal atoms. In the course of extending amidinate chemistry, we have explored a practical synthetic pathway to the alkyl-ended amidinate and *ansa*-bis­(amidinate) ligands (Bai *et al.*, 2013 ▸). They have been applied in the synthesis of Group 4 complexes, which are good catalysts for ethyl­ene polymerization (Bai *et al.*, 2010 ▸). Amidines are convenient precursors for both monoanionic amidinate ligands and bianionic *ansa*-bis­(amidinate) ligands (Coles, 2006 ▸). Some amidines could be prepared by Yb complex-catalysed addition reactions of aromatic amines and nitriles (Wang *et al.*, 2008 ▸). On the other hand, monoanionic amidinate could be used to prepare amidine and amidinium through acidolysis. Based on the same skeleton, the transformation from amidinate to amidinium will undergo an electrical inversion. Here, we report the synthesis and structural characterization of a bis­(*N*-*tert*-butyl-benzamidinium) hexa­chlorido­zirconate complex derived from the monoanionic amidinate.

## Structural commentary   

**Figure 1:**
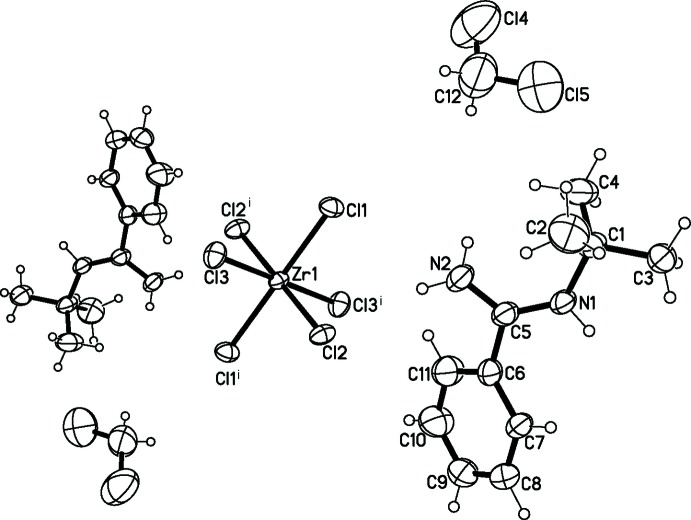
The mol­ecular structure of (I), showing the atom-numbering scheme. Displacement ellipsoids are drawn at the 30% probability level. H atoms are presented as small spheres of arbitrary radius. [Symmetry code: (i) −*x* + 2, −*y*, −*z* + 1.]

The anion in the title compound, (I), is centrosymmetric with the Zr^IV^ cation located on an inversion centre (Fig. 1 ▸) and is six-coordinated by Cl^−^ atoms. The corresponding coordination polyhedron can be described as a distorted octa­hedron where atoms Cl1, Cl2, Cl1^i^ and Cl2^i^ [symmetry code: (i) −*x* + 2, −*y*, −*z* + 1] define the equatorial plane while atoms Cl3 and Cl3^i^ occupy the axial positions. The equatorial Zr—Cl bond lengths are 2.4674 (18) Å and 2.4687 (19) Å while the axial bond length [2.433 (2) Å] is a little shorter. In the amidinium moiety, the terminal *tert*-butyl group is disposed in the direction opposite to the phenyl group on the *ipso*-carbon of the N–C–N backbone, which could minimize steric hindrance between the two groups. The dihedral angle between the aromatic ring and [NCN] plane is 43.3 (4)°. The two C—N bond lengths are equivalent [1.300 (8) and 1.299 (9) Å], composing a typical conjugated N—C—N skeleton. The lengths of the C—N bonds in (I) are shorter than those reported for a similar amidinium cation (1.325 Å; Centore *et al.*, 2003 ▸).
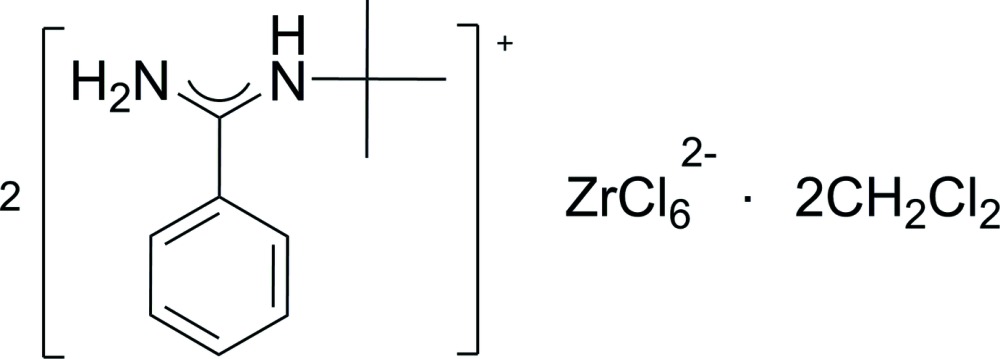


## Supra­molecular features   

**Figure 2:**
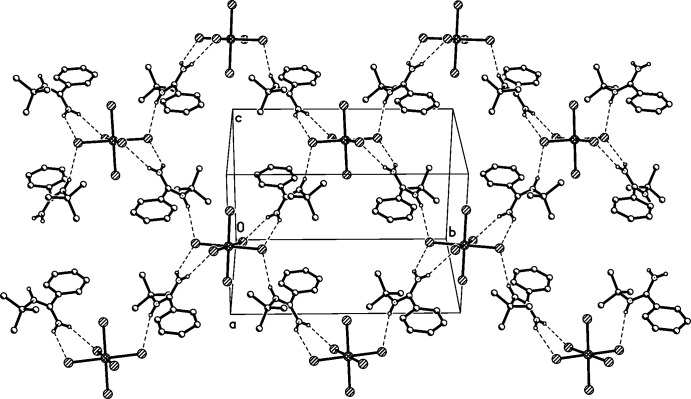
Crystal packing diagram for (I), showing the two-dimensional hydrogen-bonded network. [Symmetry codes: (ii) −*x* + 2, −*y* + 1, −*z* + 1; (iii) −*x* + 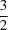, *y* + 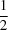, −*z* + 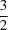.]

**Figure 3:**
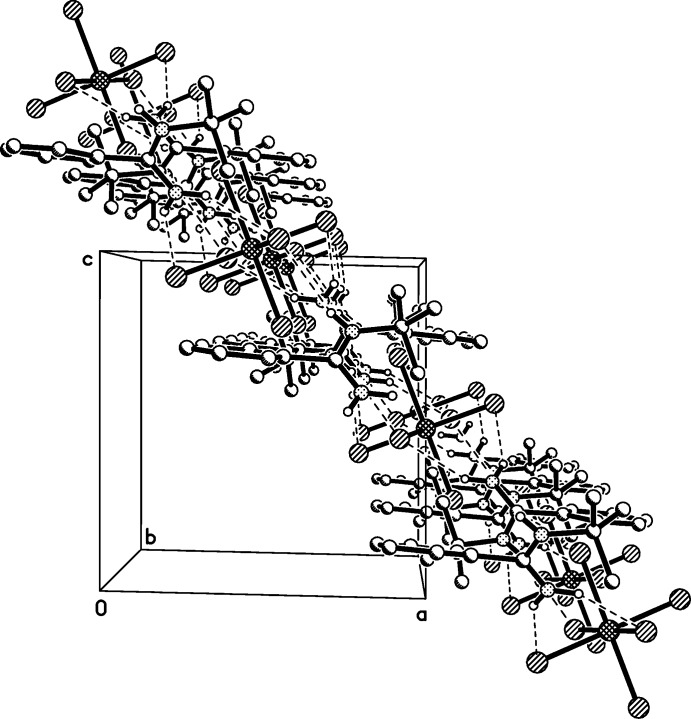
A view of the two-dimensional network along the *b* axis.

The extended structure consists of amidinium cations forming an extended hydrogen-bonded network with the chlorine atoms of the hexa­chlorido­zirconate anions. The amidinium cations involving N1 and N2 all serve as hydrogen-bond donors while only the chlorine atoms in the equatorial plane of the hexa­chlorido­zirconate anions act as acceptors (Table 1 ▸, Fig. 2 ▸). With the N—H⋯Cl hydrogen bonds, each amidinium cation connects two adjacent [ZrCl_6_]^2−^ anions and each [ZrCl_6_]^2−^ anion links four neighboring amidinium cations. The existence of bifurcated hydrogen bonds enables the formation of a two-dimensional network. Four amidinium cations and four [ZrCl_6_]^2−^ anions compose a square-like hole. [ZrCl_6_]^2−^ anions occupy the vertex positions and amidinium cations are on the edge. The corresponding motif obeys the operation of centrosymmetry and the inversion centre is the central point of the square. Moreover, the two-dimensional network extends along the *b* axis (Fig. 3 ▸). In other words, the layered network is parallel to (101) and perpendicular to (010). Besides the N—H⋯Cl hydrogen bonds, a C—H⋯Cl hydrogen bond can be observed between two centrosymmetrically related di­chloro­methane solvent mol­ecules, leading to the formation of a [CHCl]_2_ six-membered ring geometry. The angle of the C—H⋯Cl hydrogen bond is 171°, suggesting a closely linear arrangement of the related C, H and Cl atoms, also resulting in a long distance between donor and acceptor atoms [3.70 (2) Å].

## Database survey   

There are 38 structures that incorporate the zirconate anions, including [ZrCl_6_]^2−^, [Zr_2_Cl_10_]^2−^ and [Zr_2_Cl_9_]^−^. Of those 38 structures, only one has amidinium as the counter-cation (Centore *et al.*, 2003 ▸). Its [Zr_2_Cl_10_]^2−^ anion has two bridging Cl atoms and its amidinium cation has three substituents attached on the two nitro­gen atoms. In contrast to the title compound, no N—H⋯Cl hydrogen bond is observed due to the hindrance of the *N*-substituents and the lack of an N-bound hydrogen atom.

## Synthesis and crystallization   

**General Procedure**: All manipulations and reactions were performed under an inert atmosphere of nitro­gen using standard Schlenk techniques. Solvents were pre-dried over sodium, distilled from sodium-benzo­phenone (diethyl ether and dioxane) and stored over mol­ecular sieves (4 Å). CH_2_Cl_2_ was purified by distillation over CaH_2_. HCl was prepared by treating NaCl with concentrated H_2_SO_4_ and dissolved in dioxane.

**Synthesis of bis­(** ***N*** **-** ***tert*** **-butyl-benzamidinium) hexa­chlorido­zirconate(IV)**: The title compound was prepared by a one-pot reaction of *tert*-butyl­amine, LiBu, PhCN, HCl (3.6 *M* in dioxane) and ZrCl_4_. A solution of LiBu_*n*_ (2.2 *M*, 2.27 ml, 5.0 mmol) in hexane was slowly added into a solution of *tert*-butyl­amine (0.53 ml, 5.0 mmol) in Et_2_O (30 ml) by syringe at 273 K. The reaction mixture was warmed to room temperature and kept stirring for 3 h. Then benzo­nitrile (0.51 ml, 5.0 mmol) was added by syringe at 273 K. The reaction mixture was warmed to room temperature and kept stirring for 4 h. HCl (2.78 ml, 10.0 mmol, 3.6 *M* in dioxane) was added by syringe at 273 K. After stirring at room temperature for 4 h, ZrCl_4_ (0.583 g, 2.5 mmol) was added at 273 K. The resulting mixture was stirred at room temperature overnight and all volatiles were removed *in vacuo*. The residue was extracted with di­chloro­methane and the filtrate was concentrated to give colorless crystals (yield 1.325 g, 64%). The inter­mediate process involved an addition reaction of lithium amide and nitrile to yield lithium monoamidinate. Crystals of (I) suitable for single-crystal X-ray investigation were obtained by recrystallization from CH_2_Cl_2_.

## Catalytic activity for ethyl­ene polymerization   

The catalytic activity of the title compound for ethyl­ene polymerization was examined. At normal pressure and in the presence of methyl­aluminoxane (MAO), it was found to be an inactive catalyst for ethyl­ene polymerization at 303 K or higher temperature. The reaction was then performed at 10 atm in a 250 mL autoclave. However, only a trace to very small amount of polymer formation could be observed, even when heating the reaction system or changing the ratio of (I) to MAO. Therefore, a conclusion could be drawn that the title compound can not catalyse ethyl­ene polymerization.

## Refinement   

Crystal data, data collection and structure refinement details are summarized in Table 2 ▸. Hydrogen atoms were included in geometrically calculated positions. For N-bound H atoms, N—H = 0.88 Å and *U*_iso_(H) = 1.2*U*_eq_(N). For methyl­ene H atoms, C—H = 0.99 Å and *U*_iso_(H) = 1.2*U*_eq_(C) and for phenyl H atoms, C—H = 0.95 Å and *U*_iso_(H) = 1.2*U*_eq_(C). Methyl H atoms were constrained to an ideal geometry, with C—H = 0.98 Å and *U*_iso_(H) = 1.5*U*_eq_(C), but each group was allowed to rotate freely along its C—C bond.

## Supplementary Material

Crystal structure: contains datablock(s) I, global. DOI: 10.1107/S2056989016003030/xu5884sup1.cif

Structure factors: contains datablock(s) I. DOI: 10.1107/S2056989016003030/xu5884Isup2.hkl

CCDC reference: 1454857

Additional supporting information:  crystallographic information; 3D view; checkCIF report

## Figures and Tables

**Table 1:** Hydrogen-bond geometry (Å, °)

*D*—H⋯*A*	*D*—H	H⋯*A*	*D*⋯*A*	*D*—H⋯*A*
N1—H1⋯Cl2^i^	0.88	2.64	3.491 (6)	162
N2—H2*A*⋯Cl2^ii^	0.88	2.60	3.270 (7)	133
N2—H2*B*⋯Cl1^ii^	0.88	2.56	3.353 (7)	150
C12—H12*A*⋯Cl5^iii^	0.99	2.72	3.70 (2)	171

Symmetry codes: (i) 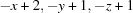; (ii) 
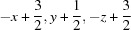
; (iii) 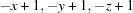.

**Table 2:** Experimental details

Crystal data	
Chemical formula	(C_11_H_17_N_2_)[ZrCl_6_]·2CH_2_Cl_2_
*M*_r_	828.30
Crystal system, space group	Monoclinic, *P*2_1_/*n*
Temperature (K)	200
*a*, *b*, *c* (Å)	10.443 (5), 16.154 (9), 10.891 (6)
β (°)	91.259 (10)
*V* (Å^3^)	1836.9 (17)
*Z*	2
Radiation type	Mo *K*α
μ (mm^−1^)	1.05
Crystal size (mm)	0.20 × 0.20 × 0.15

Data collection	
Diffractometer	Bruker SMART area-detector
Absorption correction	Multi-scan (*SADABS*; Sheldrick, 1996 ▸)
*T*_min_, *T*_max_	0.818, 0.859
No. of measured, independent and observed [*I* > 2σ(*I*)] reflections	10309, 3410, 2255
*R*_int_	0.061
(sin θ/λ)_max_ (Å^−1^)	0.606

Refinement	
*R*[*F* ^2^ > 2σ(*F* ^2^)], *wR*(*F* ^2^), *S*	0.071, 0.213, 1.00
No. of reflections	3410
No. of parameters	181
No. of restraints	1
H-atom treatment	H-atom parameters constrained
Δρ_max_, Δρ_min_ (e Å^−3^)	1.73, −0.90

Computer programs: *SMART* and *SAINT* (Bruker, 2000 ▸), *SHELXS97*, *SHELXL97* and *SHELXTL/PC* (Sheldrick, 2008 ▸).

## supplementary crystallographic information

### Crystal data

**Table:**

(C_11_H_17_N_2_)[ZrCl_6_]·2CH_2_Cl_2_	*F*(000) = 840
*M**_r_* = 828.30	*D*_x_ = 1.498 Mg m^−^^3^
Monoclinic, *P*2_1_/*n*	Mo *K*α radiation, λ = 0.71073 Å
Hall symbol: -P 2yn	Cell parameters from 2094 reflections
*a* = 10.443 (5) Å	θ = 2.3–22.0°
*b* = 16.154 (9) Å	µ = 1.05 mm^−^^1^
*c* = 10.891 (6) Å	*T* = 200 K
β = 91.259 (10)°	Block, colorless
*V* = 1836.9 (17) Å^3^	0.20 × 0.20 × 0.15 mm
*Z* = 2	

### Data collection

**Table:**

Bruker SMART area-detector diffractometer	3410 independent reflections
Radiation source: fine-focus sealed tube	2255 reflections with *I* > 2σ(*I*)
Graphite monochromator	*R*_int_ = 0.061
φ and ω scan	θ_max_ = 25.5°, θ_min_ = 2.3°
Absorption correction: multi-scan (*SADABS*; Sheldrick, 1996)	*h* = −10→12
*T*_min_ = 0.818, *T*_max_ = 0.859	*k* = −19→15
10309 measured reflections	*l* = −12→13

### Refinement

**Table:**

Refinement on *F*^2^	Primary atom site location: structure-invariant direct methods
Least-squares matrix: full	Secondary atom site location: difference Fourier map
*R*[*F*^2^ > 2σ(*F*^2^)] = 0.071	Hydrogen site location: inferred from neighbouring sites
*wR*(*F*^2^) = 0.213	H-atom parameters constrained
*S* = 1.00	*w* = 1/[σ^2^(*F*_o_^2^) + (0.1176*P*)^2^ + 2.835*P*] where *P* = (*F*_o_^2^ + 2*F*_c_^2^)/3
3410 reflections	(Δ/σ)_max_ = 0.001
181 parameters	Δρ_max_ = 1.73 e Å^−^^3^
1 restraint	Δρ_min_ = −0.90 e Å^−^^3^

### Special details

**Table:**

Geometry. All e.s.d.'s (except the e.s.d. in the dihedral angle between two l.s. planes) are estimated using the full covariance matrix. The cell e.s.d.'s are taken into account individually in the estimation of e.s.d.'s in distances, angles and torsion angles; correlations between e.s.d.'s in cell parameters are only used when they are defined by crystal symmetry. An approximate (isotropic) treatment of cell e.s.d.'s is used for estimating e.s.d.'s involving l.s. planes.
Refinement. Refinement of *F*^2^ against ALL reflections. The weighted *R*-factor *wR* and goodness of fit *S* are based on *F*^2^, conventional *R*-factors *R* are based on *F*, with *F* set to zero for negative *F*^2^. The threshold expression of *F*^2^ > σ(*F*^2^) is used only for calculating *R*-factors(gt) *etc*. and is not relevant to the choice of reflections for refinement. *R*-factors based on *F*^2^ are statistically about twice as large as those based on *F*, and *R*- factors based on ALL data will be even larger.

### Fractional atomic coordinates and isotropic or equivalent isotropic displacement parameters (Å^2^)

**Table:**

	*x*	*y*	*z*	*U*_iso_*/*U*_eq_	
Zr1	1.0000	0.0000	0.5000	0.0430 (3)	
Cl1	0.79402 (15)	0.05446 (10)	0.41944 (16)	0.0611 (5)	
Cl2	1.08156 (15)	0.14274 (9)	0.52270 (16)	0.0589 (5)	
Cl3	0.91663 (19)	0.00651 (11)	0.70660 (16)	0.0667 (5)	
N1	0.7169 (5)	0.7997 (3)	0.7141 (5)	0.0614 (15)	
H1	0.7742	0.8232	0.6673	0.074*	
N2	0.6913 (6)	0.7035 (4)	0.8664 (6)	0.0803 (19)	
H2A	0.6097	0.7159	0.8731	0.096*	
H2B	0.7259	0.6649	0.9135	0.096*	
C1	0.5840 (7)	0.8317 (4)	0.6987 (8)	0.0678 (19)	
C2	0.5393 (11)	0.8666 (7)	0.8189 (11)	0.116 (4)	
H2C	0.5848	0.8390	0.8871	0.175*	
H2D	0.4470	0.8573	0.8258	0.175*	
H2E	0.5571	0.9261	0.8219	0.175*	
C3	0.5923 (8)	0.9014 (6)	0.6076 (10)	0.097 (3)	
H3A	0.6571	0.9413	0.6360	0.145*	
H3B	0.5089	0.9290	0.6000	0.145*	
H3C	0.6161	0.8793	0.5275	0.145*	
C4	0.4977 (8)	0.7642 (6)	0.6490 (11)	0.105 (3)	
H4A	0.5305	0.7441	0.5708	0.157*	
H4B	0.4110	0.7862	0.6360	0.157*	
H4C	0.4954	0.7184	0.7079	0.157*	
C5	0.7605 (6)	0.7420 (4)	0.7870 (7)	0.0595 (17)	
C6	0.8982 (7)	0.7194 (4)	0.7806 (7)	0.0614 (17)	
C7	0.9913 (7)	0.7784 (5)	0.7678 (7)	0.070 (2)	
H7	0.9687	0.8353	0.7640	0.084*	
C8	1.1165 (8)	0.7553 (6)	0.7606 (8)	0.084 (2)	
H8	1.1813	0.7962	0.7540	0.100*	
C9	1.1485 (9)	0.6735 (6)	0.7630 (9)	0.095 (3)	
H9	1.2358	0.6577	0.7575	0.113*	
C10	1.0578 (9)	0.6150 (6)	0.7731 (11)	0.105 (3)	
H10	1.0814	0.5582	0.7733	0.127*	
C11	0.9321 (9)	0.6364 (5)	0.7831 (9)	0.092 (3)	
H11	0.8684	0.5949	0.7916	0.110*	
C12	0.335 (2)	0.4738 (14)	0.5569 (19)	0.248 (13)	
H12A	0.4220	0.4533	0.5391	0.298*	
H12B	0.3246	0.4692	0.6468	0.298*	
Cl4	0.2363 (6)	0.4119 (4)	0.4939 (5)	0.232 (3)	
Cl5	0.3316 (6)	0.5793 (3)	0.5205 (4)	0.1913 (19)	

### Atomic displacement parameters (Å^2^)

**Table:**

	*U*^11^	*U*^22^	*U*^33^	*U*^12^	*U*^13^	*U*^23^
Zr1	0.0361 (5)	0.0397 (4)	0.0535 (5)	−0.0011 (3)	0.0091 (3)	−0.0003 (3)
Cl1	0.0445 (9)	0.0580 (9)	0.0805 (11)	0.0060 (7)	−0.0031 (8)	−0.0021 (8)
Cl2	0.0490 (9)	0.0442 (8)	0.0841 (12)	−0.0066 (6)	0.0129 (8)	−0.0070 (7)
Cl3	0.0766 (12)	0.0676 (11)	0.0566 (10)	0.0072 (8)	0.0201 (8)	0.0021 (8)
N1	0.043 (3)	0.058 (3)	0.084 (4)	0.001 (2)	0.016 (3)	0.018 (3)
N2	0.057 (4)	0.075 (4)	0.110 (5)	−0.006 (3)	0.018 (3)	0.037 (4)
C1	0.045 (4)	0.056 (4)	0.102 (6)	0.002 (3)	0.015 (4)	0.015 (4)
C2	0.096 (8)	0.115 (8)	0.139 (9)	0.037 (6)	0.027 (7)	0.000 (7)
C3	0.056 (5)	0.082 (6)	0.154 (9)	0.016 (4)	0.010 (5)	0.048 (6)
C4	0.057 (5)	0.091 (6)	0.166 (10)	−0.006 (5)	−0.006 (6)	0.011 (6)
C5	0.049 (4)	0.050 (4)	0.079 (5)	−0.006 (3)	0.006 (3)	0.006 (3)
C6	0.058 (4)	0.049 (3)	0.078 (5)	0.004 (3)	0.002 (3)	0.013 (3)
C7	0.053 (4)	0.061 (4)	0.095 (5)	−0.008 (3)	0.004 (4)	0.022 (4)
C8	0.056 (5)	0.096 (6)	0.099 (6)	−0.009 (4)	0.004 (4)	0.024 (5)
C9	0.060 (5)	0.097 (7)	0.127 (8)	0.012 (5)	−0.002 (5)	0.027 (6)
C10	0.077 (6)	0.076 (6)	0.163 (10)	0.019 (5)	−0.001 (6)	0.011 (6)
C11	0.076 (6)	0.059 (5)	0.141 (8)	0.004 (4)	0.001 (5)	0.013 (5)
C12	0.25 (2)	0.30 (3)	0.191 (18)	−0.16 (2)	−0.103 (18)	0.103 (18)
Cl4	0.249 (6)	0.293 (7)	0.155 (4)	−0.105 (6)	0.044 (4)	−0.005 (4)
Cl5	0.234 (5)	0.192 (5)	0.148 (3)	0.020 (4)	0.012 (3)	−0.025 (3)

### Geometric parameters (Å, º)

**Table:**

Zr1—Cl1	2.4674 (18)	C3—H3C	0.9800
Zr1—Cl1^i^	2.4674 (18)	C4—H4A	0.9800
Zr1—Cl2^i^	2.4687 (19)	C4—H4B	0.9800
Zr1—Cl2	2.4687 (19)	C4—H4C	0.9800
Zr1—Cl3^i^	2.433 (2)	C5—C6	1.487 (10)
Zr1—Cl3	2.433 (2)	C6—C7	1.371 (10)
N1—C5	1.300 (8)	C6—C11	1.387 (10)
N1—C1	1.487 (9)	C7—C8	1.363 (11)
N1—H1	0.8800	C7—H7	0.9500
N2—C5	1.299 (9)	C8—C9	1.364 (12)
N2—H2A	0.8800	C8—H8	0.9500
N2—H2B	0.8800	C9—C10	1.345 (13)
C1—C3	1.505 (11)	C9—H9	0.9500
C1—C4	1.508 (12)	C10—C11	1.365 (13)
C1—C2	1.509 (13)	C10—H10	0.9500
C2—H2C	0.9800	C11—H11	0.9500
C2—H2D	0.9800	C12—Cl4	1.583 (17)
C2—H2E	0.9800	C12—Cl5	1.75 (2)
C3—H3A	0.9800	C12—H12A	0.9900
C3—H3B	0.9800	C12—H12B	0.9900
			
Cl3^i^—Zr1—Cl3	180.000 (1)	C1—C3—H3C	109.5
Cl3^i^—Zr1—Cl1	90.77 (7)	H3A—C3—H3C	109.5
Cl3—Zr1—Cl1	89.23 (7)	H3B—C3—H3C	109.5
Cl3^i^—Zr1—Cl1^i^	89.23 (7)	C1—C4—H4A	109.5
Cl3—Zr1—Cl1^i^	90.77 (7)	C1—C4—H4B	109.5
Cl1—Zr1—Cl1^i^	180.00 (8)	H4A—C4—H4B	109.5
Cl3^i^—Zr1—Cl2^i^	89.81 (6)	C1—C4—H4C	109.5
Cl3—Zr1—Cl2^i^	90.19 (6)	H4A—C4—H4C	109.5
Cl1—Zr1—Cl2^i^	90.07 (6)	H4B—C4—H4C	109.5
Cl1^i^—Zr1—Cl2^i^	89.93 (6)	N2—C5—N1	123.9 (6)
Cl3^i^—Zr1—Cl2	90.19 (6)	N2—C5—C6	117.8 (6)
Cl3—Zr1—Cl2	89.81 (6)	N1—C5—C6	118.3 (6)
Cl1—Zr1—Cl2	89.93 (6)	C7—C6—C11	119.5 (7)
Cl1^i^—Zr1—Cl2	90.07 (6)	C7—C6—C5	121.5 (6)
Cl2^i^—Zr1—Cl2	180.00 (8)	C11—C6—C5	118.9 (7)
C5—N1—C1	129.1 (6)	C8—C7—C6	119.9 (7)
C5—N1—H1	115.4	C8—C7—H7	120.1
C1—N1—H1	115.4	C6—C7—H7	120.1
C5—N2—H2A	120.0	C7—C8—C9	120.0 (8)
C5—N2—H2B	120.0	C7—C8—H8	120.0
H2A—N2—H2B	120.0	C9—C8—H8	120.0
N1—C1—C3	105.5 (6)	C10—C9—C8	120.6 (9)
N1—C1—C4	109.8 (6)	C10—C9—H9	119.7
C3—C1—C4	110.3 (8)	C8—C9—H9	119.7
N1—C1—C2	109.7 (7)	C9—C10—C11	120.6 (9)
C3—C1—C2	108.4 (8)	C9—C10—H10	119.7
C4—C1—C2	112.8 (8)	C11—C10—H10	119.7
C1—C2—H2C	109.5	C10—C11—C6	119.3 (9)
C1—C2—H2D	109.5	C10—C11—H11	120.4
H2C—C2—H2D	109.5	C6—C11—H11	120.4
C1—C2—H2E	109.5	Cl4—C12—Cl5	120.5 (12)
H2C—C2—H2E	109.5	Cl4—C12—H12A	107.2
H2D—C2—H2E	109.5	Cl5—C12—H12A	107.2
C1—C3—H3A	109.5	Cl4—C12—H12B	107.2
C1—C3—H3B	109.5	Cl5—C12—H12B	107.2
H3A—C3—H3B	109.5	H12A—C12—H12B	106.8

Symmetry code: (i) −*x*+2, −*y*, −*z*+1.

### Hydrogen-bond geometry (Å, º)

**Table:**

*D*—H···*A*	*D*—H	H···*A*	*D*···*A*	*D*—H···*A*
N1—H1···Cl2^ii^	0.88	2.64	3.491 (6)	162
N2—H2*A*···Cl2^iii^	0.88	2.60	3.270 (7)	133
N2—H2*B*···Cl1^iii^	0.88	2.56	3.353 (7)	150
C12—H12*A*···Cl5^iv^	0.99	2.72	3.70 (2)	171

Symmetry codes: (ii) −*x*+2, −*y*+1, −*z*+1; (iii) −*x*+3/2, *y*+1/2, −*z*+3/2; (iv) −*x*+1, −*y*+1, −*z*+1.
